# The Bioactive Potential of Algae and Their Health-Promoting Properties in the Context of the Impact on Human Health and Potential Applications in Food Technology: A Comprehensive Review

**DOI:** 10.3390/nu18213714

**Published:** 2026-11-09

**Authors:** Aleksandra Purkiewicz, Renata Pietrzak-Fiećko

**Affiliations:** Department of Commodity Science and Food Analysis, Faculty of Food Science, University of Warmia and Mazury in Olsztyn, Plac Cieszyński 1, 10-726 Olsztyn, Poland; renap@uwm.edu.pl

**Keywords:** algae, bioactive compounds, functional foods, health-promoting properties, antioxidants, polyphenols, carotenoids, polysaccharides, proteins, food technology

## Abstract

Algae are a promising source of biologically active compounds with potential significance for human health and modern food technology. Their diverse chemical composition includes, among other things, proteins and peptides, polyunsaturated fatty acids, polysaccharides, carotenoids, chlorophylls, polyphenols, vitamins, and minerals. The aim of this review is to provide a comprehensive characterization of the bioactive potential of algae and to analyze their health-promoting properties and potential applications in the production of functional foods. Particular attention was paid to compounds that have been shown in studies to exhibit antioxidant, anti-inflammatory, and antihypertensive effects, as well as those that potentially support normal glucose metabolism and have anticancer properties. The importance of selected algal components in protecting cells from oxidative stress and modulating metabolic processes was also discussed. From a food technology perspective, the possibilities for using algal biomass and components isolated from it as functional additives, natural colorants, texturizers, and sources of nutrients were presented. The potential of algae as an alternative source of protein and lipids in response to the growing demand for sustainable food raw materials was also considered. At the same time, limitations related to the bioavailability of bioactive compounds, variability in chemical composition, the presence of potential contaminants, and the need to standardize methods were highlighted. An analysis of the available data indicates that algae may be a valuable raw material for the development of functional foods; however, fully realizing their potential requires further in vivo studies and clinical trials to confirm their efficacy and safety.

## 1. Introduction

In recent years, there has been a rapid increase in interest in functional foods and naturally derived ingredients that can support health and reduce the risk of lifestyle-related diseases [1]. Changes in lifestyle, an aging population, and growing consumer awareness of the impact of diet on health are driving the search for new, valuable sources of bioactive compounds [2]. Particular attention is being paid to natural compounds with antioxidant, anti-inflammatory, immunomodulatory, anticancer, and metabolism-regulating properties, which can be used both in preventive healthcare and in the development of innovative food products [3]. Among potential sources of natural bioactive substances, algae—including both microalgae and macroalgae—are gaining increasing importance. These organisms are characterized by high nutritional value and the ability to biosynthesize a wide range of compounds with beneficial biological effects [4]. Unlike many traditional plant-based raw materials, algae can be cultivated under various environmental conditions, with relatively low water and land requirements. For this reason, they are viewed as one of the promising, sustainable sources of food ingredients for the future, aligning with the principles of a circular economy and global strategies to reduce the food industry’s environmental impact [5].

Microalgae, such as *Chlorella* spp., *Arthrospira* spp., and *Euglena* spp., are particularly valued for their high content of protein, polyunsaturated fatty acids (PUFAs), photosynthetic pigments, and other secondary metabolites [6]. Macroalgae, on the other hand—represented by brown algae (Phaeophyceae), red algae (Rhodophyta), and green algae (Chlorophyta)—are a rich source of polysaccharides such as alginates, fucoidans, laminarins, carrageenans, and agar, which are not only technologically significant but also possess potential health-promoting properties [7]. The diversity of algae’s chemical composition makes them an attractive raw material for use in the production of functional foods, dietary supplements, and innovative ingredients used in the food industry [8]. Research to date indicates that the bioactive compounds present in algae may have beneficial effects on the human body through various biological mechanisms [9]. Numerous scientific reports describe their ability to neutralize reactive oxygen species (ROS), modulate the inflammatory response, regulate lipid and carbohydrate metabolism, support immune system function, and influence the gut microbiota [8,10,11,12]. Of particular interest are compounds such as carotenoids (e.g., astaxanthin, fucoxanthin), phenolic compounds, chlorophylls, bioactive peptides, omega-3 PUFAs, and polysaccharides, which may serve as the basis for the development of new products with health-promoting properties [13].

Despite intensive research into the use of algae, their potential is still not being fully realized [14]. One of the major challenges remains the wide variation in the chemical composition of algal biomass, which depends, among other factors, on the species, environmental conditions, and the method of harvesting and processing the raw material [15]. These factors can influence the content and biological activity of individual compounds, thereby hindering the standardization of preparations and the assurance of the reproducibility of their properties. Additionally, challenges remain regarding the bioavailability of bioactive compounds, the standardization of extracts, the impact of harvesting and processing methods on biological activity, and the control of potential contaminants, such as heavy metals or toxins [16,17].

Despite numerous review articles on the biological properties of algae, their potential health benefits, and their applications in the food industry, these issues still require a critical evaluation of the available findings and their implications for the practical use of algae. The contribution of this narrative review lies in its integrated discussion of the chemical composition of microalgae and macroalgae, the main groups of bioactive compounds, and their potential health-promoting properties, including antioxidant, anti-inflammatory, antimicrobial, and metabolic activities. The potential applications of algae and their derived components in food technology are also presented, considering their impact on the nutritional value and properties of products. Particular attention was paid to the interpretation of available scientific evidence, distinguishing between in vitro studies, animal model studies, and human studies, as well as limitations related to bioavailability, standardization, and the safety of algal components. Furthermore, the article discusses technological challenges and directions for further research that could support the development of safe and sustainable food products containing algae.

## 2. Literature Search Strategy

This article presents a narrative review of the current knowledge regarding the bioactive properties of algae, their potential relevance to human health, and their potential applications in food technology. The literature search was conducted using Web of Science, Scopus, and Google Scholar databases, including publications on both microalgae and macroalgae.

Relevant publications were identified using keywords related to algal species, chemical composition, bioactive compounds, biological activities, potential health effects, and food applications. Search term included combinations of the following keywords: algae, microalgae, macroalgae, seaweed, bioactive compounds, bioactive substances, phytochemicals, health benefits, biological activity, antioxidant activity, anti-inflammatory activity, antimicrobial activity, functional food, functional ingredients, food applications, and food technology. Additional searches focused on specific groups compounds, including proteins, bioactive peptides, lipids, polyunsaturated fatty acids, polysaccharides, polyphenols, carotenoids, pigments, and antioxidants. The search terms were combined using “AND” and “OR” to refine the search results. The reference lists of relevant publications were also examined to identify the additional literature pertinent to the scope of this review.

Most of the publications included in the review were from the last 10 years, i.e., the period 2016–2026; however, earlier studies were also included if they presented significant findings relevant to the topic under discussion, formed the basis for the current state of knowledge, or concerned important research on the properties and applications of algae. The literature was selected based on its relevance to the aims and scope of the review, with particular attention to studies addressing the chemical composition of algae, the identification and characterization of bioactive compounds, their biological activities, their potential relevance to human health, and the use of algal biomass and algae-derived ingredients in food technology.

Both review articles and original experimental studies were considered. Publications concerning whole algal biomass and isolated compounds, including proteins, bioactive peptides, lipids, PUFAs, polysaccharides, polyphenols, carotenoids, and other pigments, were examined. The selected literature was critically discussed regarding the relevance of the findings, the research approaches used, the consistency of the available evidence, and the potential applications of algae in food and nutrition. As this article is a narrative review, the literature search and selection were intended to provide a broad overview of the topic rather than to constitute a systematic review or an exhaustive assessment of all available evidence. The literature search was conducted between June 2026 and September 2026. A total of 378 publications were identified and considered for this narrative review, of which 328 were selected based on their relevance to the topic and the objective of the review. The remaining publications were not considered further because they were insufficiently relevant to the subject, did not provide information relevant to the review’s objectives, lacked sufficient value, or were duplicates. The selected articles were subjected to a critical analysis in terms of their substantive value, methodological quality, the timeliness of the presented data, and their relevance to the topic under discussion.

## 3. Characteristics and Classification of Algae

Algae constitute a diverse group of photosynthetic organisms found primarily in both freshwater and marine—as well as in humid terrestrial environments [18]. This group includes both unicellular and multicellular organisms, which are primarily characterized by their ability to perform photosynthesis and use light energy to synthesize organic compounds [19]. Unlike higher plants, algae do not have typical plant organs such as roots, stems, and leaves, or conducting tissues. Algae are characterized by a wide variety of body structures ranging from microscopic unicellular forms to multicellular organisms that can reach considerable sizes [20].

### 3.1. Classification

Algae can be divided into three taxonomic groups based on their pigmentation: red algae (Rhodophyta, approximately 6000 species), brown algae (Ochrophyta, approximately 1750 species), and green algae (Chlorophyta, approximately 1200 species) [21]. The most comprehensive and widely accepted classification of algae was proposed by Fritsch (1935) [22], who divided algae into 11 groups: Chlorophyceae (green algae), Xanthophyceae (yellow-green algae), Chrysophyceae, Bacillariophyceae (diatoms), Cryptophyceae, Dinophyceae, Chloromonodineae, Euglinineae, Phaeophyceae (brown algae), Rhodophyceae (red algae), and Myxophyceae (Cyanophyceae, blue-green algae). This classification encompasses various characteristics of algae, such as pigmentation, the chemical composition of food reserves, flagellar arrangement, the presence or absence of a cell nucleus, and mode of reproduction [23].

**Figure 1:**
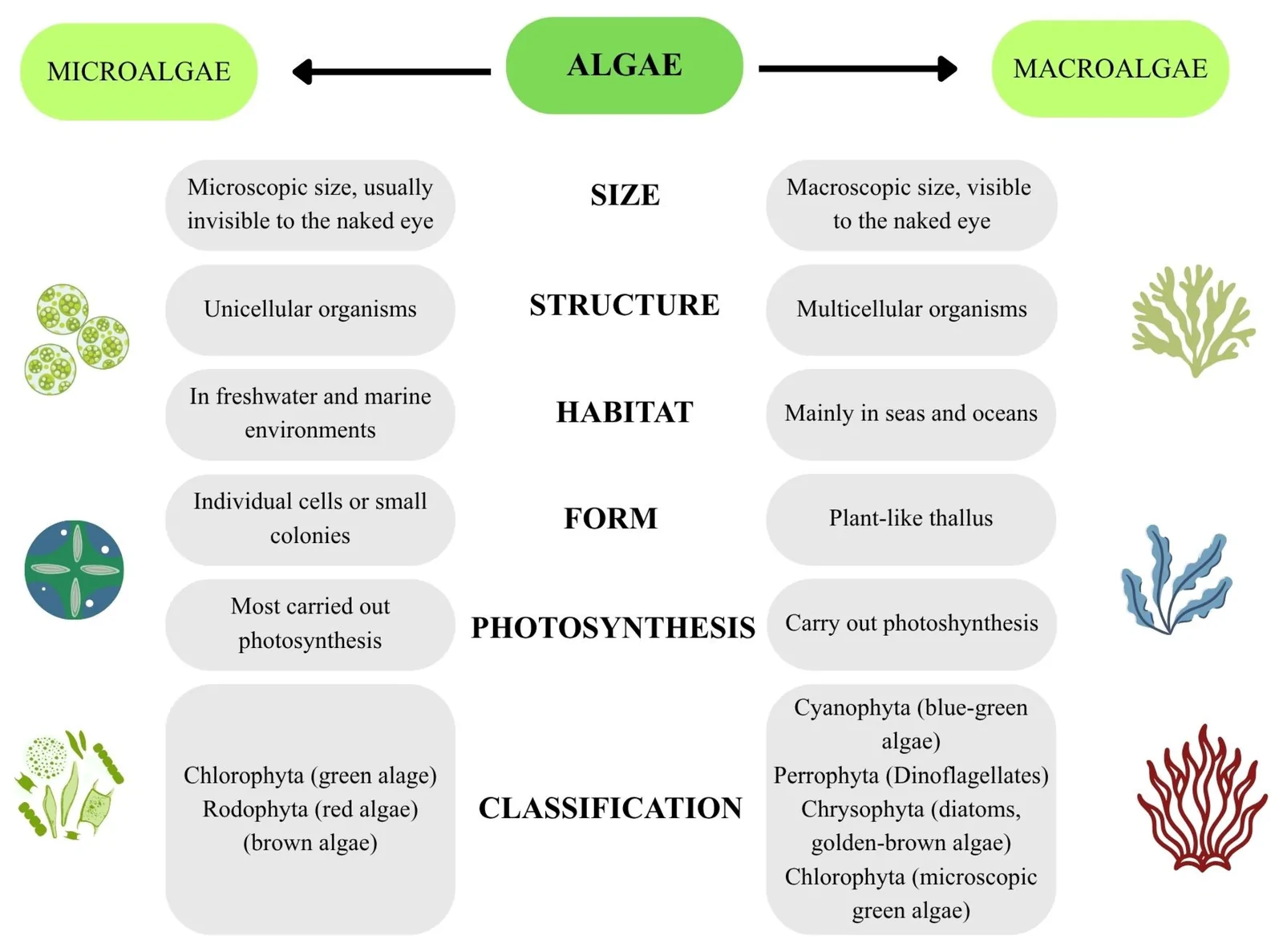
Characteristics and properties of microalgae and macroalgae.

Depending on cell size, algae can be divided into two categories: macroalgae and microalgae (Figure 1). Macroalgae are a group of multicellular organisms whose thallus can reach considerable sizes and exhibit a complex spatial organization [24]. Their morphological structure resembles that of higher plant organs, although they are not homologous to them [25]. Individual elements of the thallus perform specific functions, including those related to anchoring the organism to the substrate, absorbing nutrients, and efficiently utilizing light. Depending on the species, macroalgae can take on a wide variety of forms—ranging from simple, thin, and branched structures to elaborate thalli reaching lengths of up to several meters [7]. Macroalgae are found primarily in aquatic environments, particularly in marine ecosystems, where they form extensive communities that constitute a significant component of coastal ecosystems [26]. Due to the presence of photosynthetic pigments, they are capable of photosynthesis and thus contribute to the production of organic matter and carbon sequestration. Furthermore, macroalgae serve as a habitat and a feeding and breeding ground for numerous aquatic organisms [27,28]. Within the macroalgae, three main systematic groups are distinguished: green algae (Chlorophyta), brown algae (Phaeophyceae), and red algae (Rhodophyta). These groups differ, among other things, in the composition of their photosynthetic pigments, cellular structure, type of storage substances, and cell wall composition. These differences also affect their color, physicochemical properties, and potential for biomass utilization [16,29,30]. In green algae, the dominant pigments are chlorophylls a and b, as well as certain carotenoids—β-carotene, violaxanthin, and siphonaxanthin [31]. Algae of the class Rhodophyta contains proteinaceous pigments known as phycobiliproteins, including phycoerythrin (red) and phycocyanin (blue-green). These pigments give red algae a coloration ranging from red and reddish-brown to purple. This group of algae is also rich in carotenoids, chlorophylls a and d, and allophycocyanin [32]. Some types of red algae contain various enzymes (transferases, oxidoreductases, isomerases, and lyases) responsible for the biosynthesis of carotenoids, such as α- and β-carotene, lutein, zeaxanthin, and astaxanthin [32]. Among brown algae, the dominant pigment is fucoxanthin—a carotenoid that, depending on its concentration, imparts a color ranging from brown to yellow. Furthermore, brown algae contain chlorophylls a and c, as well as pheophytin [33]. These algal groups differ in the composition of their photosynthetic pigments, the structure and chemical composition of their cell walls, and the organization of their photosynthetic apparatus. The cell walls of green algae exhibit significant structural and chemical diversity. They typically contain cellulose microfibrils embedded in a matrix composed of glycoproteins, hemicelluloses, and pectin-like substances. In some species, additional polymers are present, which enhance the strength and resistance of the cell wall. Its composition and properties can vary depending on the species, growth phase, and environmental conditions [34]. The primary storage material is starch—composed of amylose and amylopectin—which is structurally similar to the starch accumulated by land plants [35]. The cell walls of brown algae contain cellulose microfibrils immersed in an extracellular matrix rich in polysaccharides, primarily alginates and fucoidans. Characteristic storage substances in this group include laminarin and mannitol [36]. In red algae, the cell wall contains cellulose as well as specific sulfated polysaccharides, most notably agar and carrageenans [37]. The main storage material in red algae is floridean starch, which consists primarily of amylopectin-like biopolymers and resembles glycogen [35].

The second category consists of microalgae, which mainly comprise microscopic organisms that can occur as single cells, colonies, or simple multicellular forms [38]. Unlike macroalgae, they do not form complex structures resembling the organs of higher plants [39]. Due to their small size, they typically have a high surface-to-volume ratio, which facilitates efficient exchange of substances with the environment. Microalgae inhabit both marine and freshwater environments, and some species are also found in humid and extreme environments [40,41]. Microalgae include both prokaryotic and eukaryotic organisms. Based on taxonomic classification, they include, among others, cyanobacteria and eukaryotic microalgae belonging to various groups, such as Chlorophyta (green algae), Diatoms, and Dinophyta. The taxonomic diversity of microalgae is associated with varying profiles of the bioactive compounds they produce and their potential biological significance [42].

### 3.2. Nutritional Composition

#### 3.2.1. Microalgae

Microalgae are a good source of protein, fats, and carbohydrates, and they also contain a range of vitamins and minerals [43]. Their composition varies depending on the species and growth conditions. A short-term drop in temperature causes an increase in carbohydrate content and a decrease in lipids in certain green microalgae. These changes are reversible and depend on the species and age of the culture [8].

Microalgae are valued primarily for their high protein content. It has been reported that *Athrospira plantesis* and *Chlorella vulgaris* contain up to 70% dry protein. Furthermore, the amino acid profile of selected algal species is comparable to that of eggs [44,45]. Among vitamins, microalgae are richest in B vitamins (B12, B1, B2, B3, B6), but they also contain vitamin C and the fat-soluble vitamins A and K [43]. Studies have reported the presence of biologically active vitamin B_12_ in *C. vulgaris*, suggesting its potential as a vegetarian source of this vitamin. However, vitamin B_12_ content may vary depending on cultivation conditions and the characteristics of the biomass, and its contribution to meeting human dietary requirements requires further consideration [46,47].

Microalgae are a valuable source of lipids, the content of which varies widely—from 1 to 70% of dry biomass—and depends, among other factors, on species, temperature, light, pH, and nutritional conditions [48,49]. They consist primarily of monounsaturated and polyunsaturated fatty acids, including essential omega-3 and omega-6 fatty acids such as α-linolenic acid (ALA), eicosapentaenoic acid (EPA), docosahexaenoic acid (DHA), linoleic acid (LA), γ-linolenic acid (GLA), and arachidonic acid (AA) [49]. Individual species differ in their PUFA content—*Crypthecodinium cohnii* contains mainly DHA, *Nannochloropsis oculata* and *Phaeodactylum tricornutum* are rich in EPA, while *Porphyridium. cruentum* is rich in AA [50]. Among microalgae, the species richest in fat include *Schizochytrium* sp., *Nannochloropsis* sp., and *C. vulgaris* [49]. Furthermore, *Auxenochlorella protothecoides* can contain up to 70% lipids, making it a promising source of fatty acids that can be used for nutritional and nutraceutical purposes [49].

Microalgae are an important source of carbohydrates, mainly in the form of polysaccharides, the content and composition of which depend on the species, strain and culture conditions. Polysaccharides can be divided into three main groups: structural, building cell walls; backup, serving as energy and coal storage; and extracellular. The cell walls of microalgae show significant diversity in terms of structure and composition, and the polysaccharides present in them include, among others: β-glucans, β-mannans, α-rhamnans and β-galactofuranans. Depending on the species, their structural components may also be cellulose or chitin [51,52].

It has been noted that the minerals found in microalgae are more easily absorbed than those from higher terrestrial plants. This is due to the pigment phycocyanin, which, during digestion, forms soluble complexes with iron and other minerals, thereby facilitating their absorption and increasing their bioavailability [43]. Microalgae are a good source primarily of iodine, potassium, iron, magnesium, and calcium [53].

Microalgae are a rich source of pigments—chlorophylls [49] and carotenoids such as fucoxanthin, astaxanthin, lutein, zeaxanthin, canthaxanthin, and β-carotene [43]. These compounds primarily exhibit antioxidant properties, as well as potential anti-inflammatory, anticancer, and antidiabetic effects. Fucoxanthin and astaxanthin are particularly valuable pigments, and their content may increase under stressful conditions. In addition, microalgae contain phenolic acids, including gallic, chlorogenic, ferulic, cinnamic, and coumaric acids, as well as flavonoids, including quercetin, catechin, and kaempferol [49]. Their presence has been confirmed, among others, in the species *Spirulina*, *Chlorella*, and *Nannochloropsis* [54,55]. Phenolic compounds exhibit potential antioxidant and anti-inflammatory effects, making microalgae a good source of ingredients for nutritional and health-promoting applications [49].

#### 3.2.2. Macroalgae

As with microalgae, macroalgae are an excellent source of nutrients, the composition of which varies depending on the time and location of harvest [43]. However, their composition is strongly dependent on the type of macroalgae. Red algae and green algae contain significantly higher levels of protein compared to most brown algae [56]. The protein content in macroalgae can range from 7 to 31% of dry weight [24]. High-protein seaweed species can be used as ingredients in a variety of food products. An example is the Porphyra species, commonly used in the production of nori and other food products in Japan [57]. *Ulva pertusa*, *Enteromorpha*, and *Monostroma* also have high protein content, reaching approximately 20–26% of dry weight. In Europe and Canada, *Palmaria palmata* is a popular source of protein, with a protein content that can reach up to 35% of dry weight [24].

Macroalgae are characterized by a relatively low lipid content, typically less than 5% of dry weight. Their quantity and composition depend on the species and environmental conditions, such as temperature, salinity, and light intensity [24]. Brown algae generally contain more lipids than green algae, and a significant portion of these lipids consists of PUFAs, particularly EPA and ARA. Red and brown algae are particularly rich in these acids, while green algae contain, among others, linoleic acid and α-linolenic acid. The lipid fraction of macroalgae also includes glycolipids and phospholipids, which perform important structural functions and may exhibit biological activities, including anti-inflammatory effects [24,58].

The carbohydrate content in macroalgae can vary widely and reach up to 76% on a dry weight basis [24]. Species particularly rich in carbohydrates include *Gracilaria chilensis* (66.1% of dry weight) and *Saccharina japonica* (51.9% of dry weight) [24]. An important group consists of polysaccharides such as alginates, carrageenan, fucoidans, laminarin, and ulvans, which exhibit potential antioxidant, anti-inflammatory, anticoagulant, and neuroprotective properties [59]. Their biological activity depends, among other things, on their chemical structure, molecular weight, and degree of sulfation. Polysaccharides also perform important structural functions in the cell walls of various groups of algae. Due to their gelling and thickening properties, compounds such as agar, alginates, and carrageenan are widely used in the food industry [24].

The chemical composition of selected micro- and macroalgal species is summarized in Table 1.

## 4. Bioactive Compounds in Algae

### 4.1. Phenolic Compounds

Among the metabolites present in algae, phenolic compounds—which are characterized by great structural diversity—hold a special place [71]. In algal cells, phenolic compounds can occur in various forms, both as individual molecules and in combination with other cellular components [72]. The concentration of polyphenols in algae depends on the season, habitat, as well as exposure to UV radiation, light, and the availability of nutrients and salinity levels [73]. The groups of phenolic compounds detected in algae primarily include phenolic acids, flavonoids, tannins, and tannic acids [74]. Bromophenols, phenolic acids, and flavonoids predominate in green algae and red algae. In brown algae, on the other hand, florotanins are the main components [54].

Bulut et al. [75] demonstrated that *Scenedesmus* sp. extracts differed in their phenolic compound content depending on the solvent used, with the highest value observed for the ethanol–water extract. The extraction method influences the recovery and measured content of phenolic compounds in algal extracts [76]. Brown algae are considered important sources of polyphenolic compounds, particularly phlorotannins, and have been reported to exhibit higher antioxidant activity than red and green algae [77]. However, phlorotannin content varies considerably among species. For example, in brown algae collected off Peniche, western Portugal, the reported phlorotannin content ranged from 288.20 µg/g in *Cystoseira usneoides* to 815.24 µg/g in *Cystoseira tamariscifolia*. A higher value was reported for *Fucus spiralis* (968.57 µg/g), whereas *Sargassum vulgare* contained 74.96 µg/g [78]. These findings highlight substantial interspecific variation in phlorotannin content and the importance of considering species identity when comparing the polyphenolic profiles of brown algae [77,78].

### 4.2. Terpenoids

Terpenoids belong to one of the most diverse groups of naturally occurring metabolites. Their structure is based on isoprene units, and the complexity of individual compounds depends primarily on the number of these units and the type of chemical modifications that occur. Processes such as oxidation result in numerous derivatives that differ in their properties and biological activity. Based on the number of building blocks, the group is classified into, among others, mono-, di-, and sesquiterpenoids, as well as triterpenoids and tetraterpenoids [79].

One of the most important groups of terpenoids found in algae are carotenoids. These compounds are present in both microalgae and macroalgae, where they participate in processes related to photosynthesis and protect cells from the effects of oxidative stress [73].

Among the carotenes found in algae are, among others, α-carotene, β-carotene, and lycopene. In turn, examples of xanthophylls include astaxanthin, lutein, canthaxanthin, violaxanthin, zeaxanthin, and fucoxanthin [80]. Astaxanthin, produced by *Haematococcus pluvialis*, is one of the most potent natural antioxidants and is widely used in nutraceuticals and cosmetic products. Fucoxanthin, on the other hand, which is found in large quantities in diatoms such as *P. tricornutum* and *Odontella aurita*, exhibits potential anti-obesity, anti-inflammatory, and anticancer effects [81].

### 4.3. Fatty Acids

Lipids, including fatty acids, are also an important component of algal biomass; they contribute to the structure of cell membranes and perform functions related to metabolic processes [82].

Among the saturated fatty acids (SFAs) found in algae are palmitic, myristic, and stearic acids, while monounsaturated fatty acids (MUFA) include oleic acid. From a nutritional standpoint, polyunsaturated fatty acids are particularly important, especially those belonging to the omega-3 family [83].

Some species of microalgae can synthesize long-chain polyunsaturated fatty acids (LCPUFAs), including EPA and DHA. These fatty acids have been detected, among others, in microalgae such as *P. tricornutum*, *N. oculata*, *Seminavis gracilenta*, *Isochrysis galbana*, and *Prorocentrum minimum* [84]. Due to their content of these acids, certain species of microalgae are considered an alternative source of valuable lipids that can be used in the production of foods with enhanced nutritional and functional value.

### 4.4. Polysaccharides

Eukaryotic microalgae synthesize polysaccharides whose structure is based primarily on simple sugars such as xylose, galactose, glucose, rhamnose, mannose, fucose, and fructose [85]. In turn, macroalgae contain, among other things, alginate, laminarin, fucoidan, agarose, porphyran, carrageenan, and ulvan [86].

Sulfated polysaccharides are attracting significant research interest due to the wide range of biological activities attributed to them. They are noted for their antioxidant, anti-inflammatory, antimicrobial, antiviral, immunomodulatory, and anticoagulant potential, as well as their potential use as prebiotic ingredients [87,88]. Particular attention is drawn to fucoidans—found primarily in brown algae—and ulvans and carrageenans—found in green and red algae—whose activity is linked, among other factors, to the presence of sulfate groups [89,90]. Mechanisms of antioxidant activity may include, among others, the direct neutralization of ROS, metal ion chelation, and the modulation of endogenous antioxidant defense systems [88].

The biological properties of algal polysaccharides also depend on their molecular weight and structural characteristics, such as monosaccharide composition, the type of glycosidic bonds, the degree of branching, and the degree and distribution of sulfate groups [91,92]. Certain algal polysaccharides, including unsulfated alginate and laminaran, are utilized by specific microorganisms as fermentation substrates. This process can lead to the production of short-chain fatty acids, which play a role in regulating the intestinal environment and supporting the integrity of the intestinal barrier. These effects depend on the structure of the polysaccharide and the composition of the microbiota [93].

Marine microalgae polysaccharides have potential antiviral, antimicrobial, antioxidant and immunomodulatory properties. In vitro studies on intestinal cells and in vivo studies on mice have shown that microalgae polysaccharides may have antioxidant and anti-inflammatory effects and support the protection of the intestinal barrier [94].

### 4.5. Bioactive Peptides

**Figure 2:**
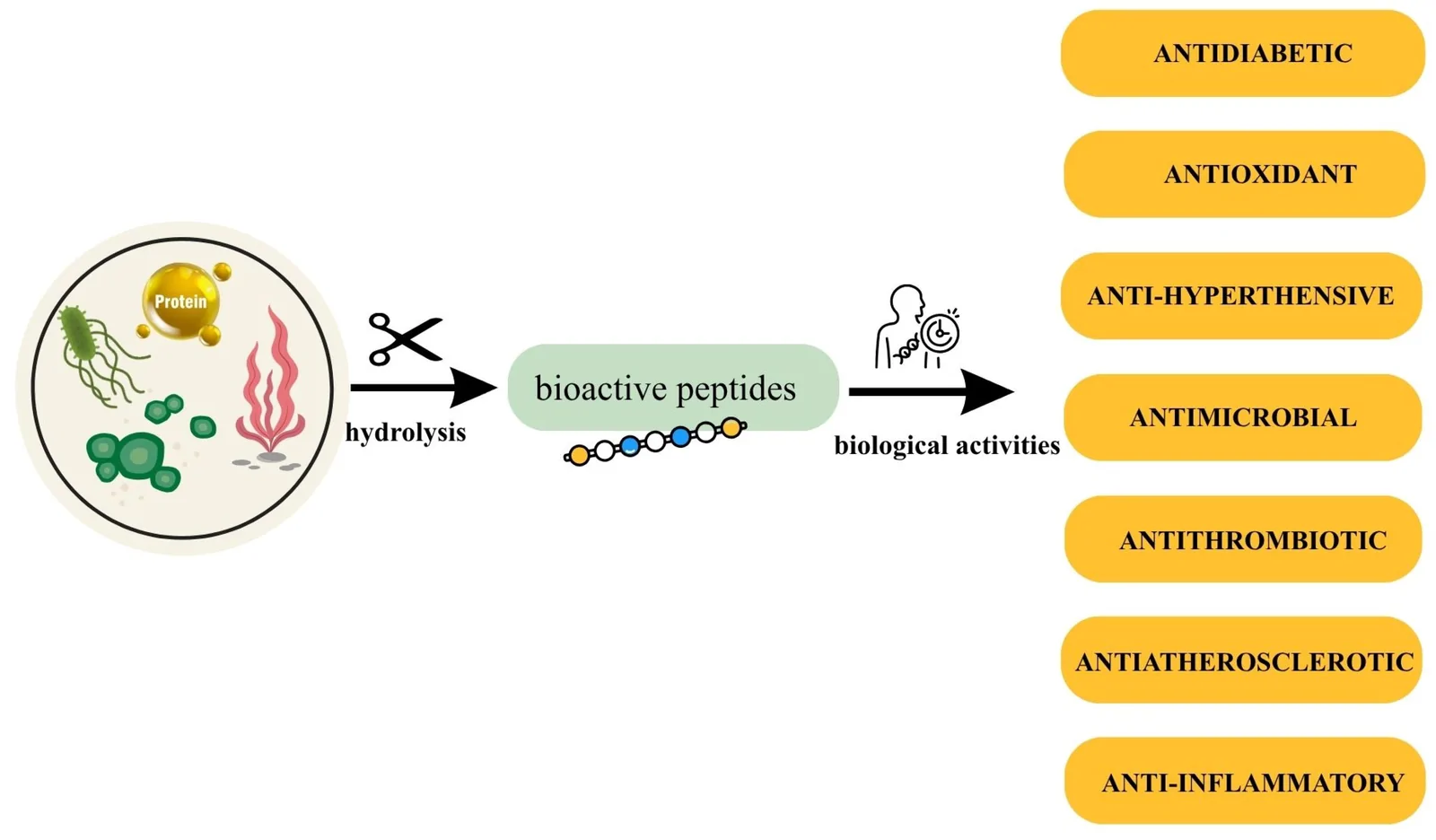
Biological activities of algal peptides.

Algal proteins are a valuable source of bioactive peptides, which, once released from their parent proteins, may exhibit specific physiological effects [95]. Research to date indicates that bioactive peptides derived from algae may exhibit, among other effects, antioxidant, antihypertensive, anti-inflammatory, antimicrobial, antidiabetic, immunomodulatory, anticancer effects, and may also inhibit angiotensin-converting enzyme (ACE) (Figure 2) [96,97]. Other peptides may affect enzymes involved in carbohydrate metabolism, such as α-amylase and α-glucosidase, which is being studied in the context of their potential to aid in glycemic control [98].

Although many bioactive peptides with potential health-promoting effects, including antihypertensive properties, have been isolated from macro- and microalgae, their industrial-scale production and commercialization still pose numerous technological challenges that require further research and the development of appropriate solutions [96].

### 4.6. Photosynthetic Pigments

Photosynthetic pigments constitute a significant group of bioactive compounds found in algae and are responsible for absorbing light energy and utilizing it in the process of photosynthesis [99]. Their composition and content are characteristic of specific groups of algae. Green algae contain primarily chlorophylls a and b as well as carotenoids; brown algae contain chlorophylls a and c and the characteristic pigment fucoxanthin; while red algae contain chlorophyll a and phycobiliproteins, primarily phycoerythrin and phycocyanin [100].

Beyond their photosynthetic function, these pigments exhibit significant biological potential, primarily linked to their antioxidant properties. Chlorophyll and its derivatives, including pheophytin, may exhibit anti-inflammatory effects. These properties have been observed in animal studies, such as in a model of carrageenan-induced paw edema in mice and formalin-induced inflammation in rats [101]. Furthermore, it has been shown that certain chlorophyll derivatives can mitigate oxidative DNA damage and lipid peroxidation induced by hydrogen peroxide; they can also bind Fe^2+^ ions, which may help limit oxidative processes [101]. In vitro studies have also demonstrated that chlorophyll-related compounds can contribute to reducing the levels of ROS—which, in excessive amounts, can damage lipids, proteins, and DNA [102,103]. The literature also describes the potential antibacterial properties of chlorophyll and its derivatives, as well as their possible influence on the composition of the gut microbiota. In studies involving mice, changes were observed in the proportions of bacteria belonging to the phyla Firmicutes and Bacteroidetes—specifically, a decrease in the abundance of the former and an increase in the latter [104]. Research is also being conducted on the potential anti-cancer properties of chlorophyll. Current findings suggest, among other things, the ability to inhibit the activation of certain carcinogens, modulate antioxidant processes, and induce apoptosis in cancer cells [105]. These mechanisms are the subject of ongoing research and do not constitute proof of the efficacy of chlorophyll in the treatment of cancer. Chlorophylls are also used in the food industry—not merely as colorants, but as potential ingredients that enhance the functional value of products. One study demonstrated that enriching crackers with biomass from the algae *A. platensis* and *C. vulgaris* increased their antioxidant potential. This finding suggests that algal biomass could be used as an ingredient to fortify food with compounds possessing antioxidant properties [106].

Carotenoids, including fucoxanthin, can neutralize ROS and limit oxidative processes within cells. The literature also describes their potential anti-inflammatory, antidiabetic, and antimicrobial effects, as well as their role in modulating lipid metabolism [107]. Research indicates that it may have potential anticancer effects; however, the findings are not always conclusive. For example, fucoxanthinol, a metabolite of fucoxanthin, has been shown in some studies to inhibit the growth of cancer cells and induce their apoptosis [108], while other studies have shown that it can stimulate the growth of human pancreatic cancer cells [109]. Fucoxanthin also exhibits antioxidant potential, which has been studied in the context of protecting cells from oxidative stress. In vitro studies have shown that it can reduce lipid peroxidation and ROS production in various cell types, including by influencing the activity of antioxidant enzymes such as catalase. Some studies have also demonstrated its potential to reduce DNA damage and preserve normal cell morphology [110]. Furthermore, fucoxanthin exhibits potential anti-inflammatory effects. Animal studies have also shown a reduction in inflammation in adipose tissue and the liver, as well as protection of the skin against damage caused by UVB radiation [111]. Another study demonstrated that fucoxanthin supplementation may support weight loss and the reduction in fatty liver, lower markers of inflammation, and improve metabolic parameters in patients with nonalcoholic fatty liver disease (NAFLD) or obesity, with no significant adverse effects observed [112,113]. Phycobiliproteins—particularly phycoerythrin and phycocyanin—also constitute a significant group; in addition to their role in photosynthesis, they exhibit antioxidant properties and potential anti-inflammatory activity [114]. C-phycocyanin and phycoerythrin exhibit strong antioxidant properties, stemming from the presence of chromophores capable of directly neutralizing ROS [115]. Furthermore, the amino acid composition of phycobiliproteins—characterized by a significant content of glutamic acid, glycine, and arginine—may facilitate the binding of metal ions and the neutralization of ROS [116]. Phycobiliproteins can protect neurons against oxidative stress, support mitochondrial function, and mitigate changes associated with Parkinson’s and Alzheimer’s diseases [115]. Phycocyanin may also reduce lipid peroxidation and contribute to the protection of myelin and nerve fibers in models of multiple sclerosis [117]. Phycobiliproteins, particularly C-phycocyanin, demonstrate anti-inflammatory effects by limiting the production of pro-inflammatory cytokines [118]. They may also reduce pain and alleviate intestinal inflammation, helping to protect the mucous membrane. Additionally, they influence the immune system, for instance by regulating the activity of macrophages and lymphocytes [115]. Research has also shown that they may mitigate allergic reactions by reducing the release of histamine [119]. Their potential benefits in neurological conditions—linked to the reduction in inflammation and oxidative stress—are also being investigated [115]. However, most of these effects have been observed in laboratory studies and animal models, meaning further clinical research is required. Regarding anti-cancer properties, phycocyanin may inhibit the development of cancer cells by inducing programmed cell death. It can also suppress inflammation that promotes tumor progression, potentially hindering tumor growth and further development [120].

## 5. Health-Promoting Properties

### 5.1. Antioxidant Properties

Algae are a rich source of numerous biologically active compounds, such as polysaccharides, polyphenols, carotenoids, phycobiliproteins, sterols, peptides, vitamins, minerals, and PUFAs [121]. In recent years, algal biomass and algae-derived metabolites have attracted increasing research interest because of their potential relevance to chronic diseases, including cardiovascular, metabolic, neurodegenerative, and cancer-related conditions [9]. However, further research is needed to better understand the health-promoting properties of algae and their potential applications. The health-promoting properties of algae stem primarily from their ability to modulate processes related to oxidative stress, inflammation, immune system function, and metabolic regulation [122].

One of the main mechanisms of action is the direct scavenging of ROS. Bioactive compounds present in algae, especially phenolic compounds—including flavonoids and tannins—can neutralize free radicals by donating electrons or hydrogen atoms, thereby limiting oxidative damage to cells. In addition, they bind metal ions, thereby limiting the possibility of the Fenton reaction, which produces the highly toxic hydroxyl radical [123].

The activation of the Nrf2 (nuclear factor erythroid 2-related factor 2) pathway also plays a significant role. Nrf2 is a transcription factor that, following its translocation to the cell nucleus, binds to antioxidant response elements (ARE), regulating the expression of genes involved in the cellular response to oxidative stress, including genes encoding antioxidant enzymes such as catalase (CAT), superoxide dismutase (SOD), and glutathione peroxidase (GPx), as well as other protective molecules, including heme oxygenase-1 (HO-1). Activation of the Nrf2 pathway thus enhances the endogenous antioxidant protection of cells [123]. Experimental studies suggest that fucoxanthin and other bioactive compounds from brown algae may exert antioxidant effects. In some experimental models, these effects have been associated with activation of the Nrf2/HO-1 pathway, increased expression of antioxidant-related genes, and reduced intracellular ROS levels [110,124,125]. Experimental studies indicate that fucoxanthin may also affect the activity of antioxidant enzymes, including SOD, CAT, and GPx [126]. In an animal model of ovalbumin-induced asthma, fucoxanthin treatment was associated with reduced ROS production and increased antioxidant enzyme activity [127]. Similarly, in a rat model of lipopolysaccharide (LPS)-induced uveitis, fucoxanthin increased SOD activity and decreased malondialdehyde (MDA) levels, a marker of lipid peroxidation [128]. These findings provide evidence of antioxidant effects in specific animal models but do not establish comparable effects in humans.

### 5.2. Anti-Inflammatory Properties

Inflammation is the body’s natural defense response to harmful factors, including injuries and pathogens. Mediators such as IL-1 and IL-6 (interleukins), TNF-α (tumor necrosis factor-α), and PGE2 (prostaglandin E2), as well as the enzymes COX-2 (cyclooxygenase-2) and iNOS (inducible nitric oxide synthase) [129]. Although inflammation serves a defensive function, its chronic nature may contribute to the development of, among other conditions, obesity, type 2 diabetes, atherosclerosis, cancer, and neurodegenerative diseases. Experimental studies indicate that selected algae-derived compounds may modulate inflammatory pathways and reduce the production of pro-inflammatory mediators [9].

The anti-inflammatory activity of algae is determined not only by their species, but also by the chemical composition and properties of the bioactive compounds they contain. Among the substances with anti-inflammatory potential, phlorotannins—polyphenols characteristic of brown algae—play a significant role; they can modulate the NF-κB and MAPK signaling pathways, which are involved in regulating the inflammatory response. Phlorotannins also inhibit enzymes that participate in the production of pro-inflammatory mediators (such as phospholipase A2, lipoxygenases, and cyclooxygenase-2) [130,131,132]. Polysaccharides are also important, including fucoidan—found primarily in brown algae—and ulvan, which is characteristic of green algae. These compounds can influence immunological processes and the course of an inflammatory reaction, for instance by limiting the production of pro-inflammatory mediators and modulating signaling pathways associated with the inflammatory response [133,134]. However, their biological activity depends on their structural properties—such as molecular weight and the degree of sulfation—as well as the concentration used [135].

The therapeutic potential of algae also stems from the presence of numerous bioactive compounds, such as polysaccharides, proteins, sterols, terpenoids, polyphenols, and cyclic polysulfide compounds. These compounds may support gut health by modulating the microbiota, improving intestinal barrier function, and regulating the immune response. Due to these properties, algae are currently being studied as a potential source of substances that may help alleviate inflammation in inflammatory bowel disease (IBD). Nevertheless, evidence from experimental studies is insufficient to establish their efficacy as a treatment for IBD in humans [12].

In vitro and animal studies have reported anti-inflammatory effects of extracts from several marine algal species, including reduced expression of iNOS (inducible nitric oxide synthase) and COX-2 (cyclooxygenase-2) and lower production of mediators such as (nitric oxide), TNF-α (tumor necrosis factor-α), IL-1β, and IL-6 (interleukins) [83,136,137]. In a mouse model of DSS-induced colitis, a *C. vulgaris* extract was associated with reduced disease-related changes and increased levels of regulatory T cells [138]. Similarly, caulerpin, a compound isolated from algae of the genus Caulerpa, reduced the levels of several pro-inflammatory cytokines and was associated with less colon damage and shortening in experimental models [139,140].

These examples indicate that the anti-inflammatory activity of algae may involve both the reduction in pro-inflammatory mediator production and the modulation of the immune response. However, it is not possible to definitively identify a single algal species as the most effective, as the studies presented differ in terms of the experimental models used, the dosages applied, and the parameters evaluated.

In a cell-based study, lipid extracts from *Pavlova lutheri* and *P. palmata* inhibited LPS-induced production of IL-6 (interleukin 6) in THP-1 macrophages, while the extract from *P. palmata* also reduced IL-8 (interleukin 8) production. This effect may be related to the presence of PUFAs, particularly EPA, and bioactive pigments such as chlorophyll a, β-carotene, and fucoxanthin. All the extracts studied also affected the expression of genes associated with the inflammatory response [141].

The biological activity of algal preparations can also be influenced by the methods used to process them. Fermentation plays a significant role; it can modify the chemical composition of algae and enhance the anti-inflammatory activity of the resulting preparations. Enzymes produced by microorganisms break down complex polysaccharides into smaller molecules, which may exhibit prebiotic and anti-inflammatory effects. For instance, oligosaccharides derived from algae can be utilized by gut bacteria to produce short-chain fatty acids, which support proper intestinal function and help regulate the inflammatory response [93,142]. A study on *Sargassum thunbergii* demonstrated that fermentation with *Lactobacillus* sp. SH-1 reduced nitric oxide production and downregulated the expression of specific pro-inflammatory factors in cells exposed to an inflammatory stimulus [143]. These findings suggest that fermentation may enhance the anti-inflammatory potential of certain algal preparations under experimental conditions. However, the effects depend on the specific conditions applied and warrants further research [144].

### 5.3. Antidiabetic Activity

Diabetes is a chronic metabolic disease characterized by hyperglycemia and disturbances in the metabolism of carbohydrates, fats, and proteins. Its development results from insulin deficiency, impaired insulin action, or reduced tissue sensitivity to insulin [145]. Algae are being investigated as potential sources of bioactive compounds that may support the regulation of glucose metabolism through various mechanisms, including the inhibition of carbohydrate digestion, the improvement of glucose utilization, and the reduction in oxidative stress and inflammation [145].

One of the mechanisms behind the potential anti-diabetic effect of algae is the inhibition of enzymes involved in carbohydrate digestion. A butanol extract from Spirulina inhibited α-glucosidase activity, achieving an IC50 value of 23 μg/ml [146]. A similar effect was observed for the exopolysaccharides of *Pseudanabaena* sp., whose extract inhibited α-glucosidase activity by 14.02% [147]. In turn, fucoxanthin isolated from *P. tricornutum* exhibited concentration-dependent inhibition of α-amylase and α-glucosidase, with IC50 values of 0.68 and 4.75 mmol/L, respectively [148]. In addition, extracts from the brown macroalga *Sargassum polycystum* may reduce postprandial hyperglycemia by inhibiting intestinal glucose absorption. This points to the potential of extracts from brown macroalgae as natural alternatives to synthetic enzyme inhibitors, such as acarbose, whose use may be associated with gastrointestinal side effects [149].

The potential effects of algae on glucose metabolism have been investigated in both experimental and human studies. In vitro studies demonstrated that methanolic and 80% methanolic extracts of the brown algae *Polycladia myrica* and *Padina antillarum* exhibited inhibitory activity against α-glucosidase, an enzyme involved in carbohydrate digestion. These findings suggest that bioactive compounds present in these algae may help regulate carbohydrate breakdown and contribute to their potential antidiabetic effects [150]. In a study involving 20 healthy adults, consumption of *Laminaria digitata* and *Undaria pinnatifida* with a starch-containing meal reduced postprandial glucose and insulin responses [151]. Furthermore, a randomized clinical study involving 60 patients with type 2 diabetes reported improvements in glycemic control and lipid parameters following supplementation with *A. platensis* (2 g/day) alongside metformin for three months [152]. Although these findings are promising, the studies differ in population, intervention, and outcome measures. They therefore do not establish that algae in general are effective in preventing or treating diabetes.

It should be emphasized that diabetes is also associated with oxidative stress and chronic inflammation [149]. Therefore, the antioxidant and anti-inflammatory properties of algae may be relevant to metabolic health. However, further clinical studies are needed to establish their efficacy and safety in the prevention and treatment of diabetes.

### 5.4. Anti-Obesity Effects

Obesity is a metabolic disorder characterized by excessive accumulation of adipose tissue and a BMI > 30 kg/m^2^. It poses a global threat to public health and increases the risk of developing type 2 diabetes, heart and liver diseases, and dyslipidemia [153]. Treatment for excess body weight involves weight reduction through improved dietary habits and the incorporation of physical activity into daily routines. In cases of high BMI and the presence of conditions associated with obesity, pharmacological interventions are increasingly being implemented to aid in the treatment of excess weight [153]. Due to the side effects associated with anti-obesity medications, there is growing interest in identifying natural substances that may prove effective in treating excess body weight [153].

One of the therapeutic goals in treating disorders related to excessive fat accumulation is the inhibition of pancreatic lipase activity [153]. It has been shown that available drugs with this mechanism of action may be associated with, among other things, impaired absorption of certain vitamins and increased fat content in stool [154,155,156]. One study from 1999 examined 54 species of marine algae and demonstrated high pancreatic lipase-inhibiting activity for *Caulerpa taxifolia* and *Asparagopsis taxiformis*, reaching nearly 100%. Promising activity was also observed for *Codium latum*, *Gloiopeltis tenax*, *Hypnea charoides*, *Sargassum muticum*, and *Dictyopteris latiuscula*. Caulerpenin was isolated from a Caulerpa taxifolia extract; when administered to rats, it reduced and delayed the increase in blood triacylglycerol levels [157].

Alginates found in algae also exhibit high anti-obesity potential. Alginates derived from *Laminaria hyperborea*, characterized by a high glucuronic acid content, exhibited a stronger inhibitory effect than alginates from *Lessonia nigrescens*, which are rich in mannuronic acid [158]. The inhibitory activity of these alginates persisted after heat treatment and under simulated intestinal digestion conditions, suggesting their potential as food ingredients for further investigation into the modulation of fat digestion and absorption [159]. Whether these effects translate into clinically meaningful changes in body weight in humans remains to be established.

In addition to its antioxidant and antidiabetic properties, fucoxanthin has a documented anti-obesity effect. In animal studies, fucoxanthin and conjugated linoleic acid (CLA) complex liposomes showed potential anti-obesity effects by regulating AMP-activated protein kinase (AMPK) signaling, influencing lipid metabolism, reducing fat accumulation, and promoting energy balance in mice feed a high-fat diet [160]. In another study, in vitro experiments showed that a fucoxanthin-rich *P. tricornutum* extract reduced lipid accumulation in adipocytes, while in vivo experiments demonstrated reduced weight and fat mass in mice fed a high-fat diet [161]. These results suggest potential metabolic effects in the animal models studied but do not establish efficacy for weight management in humans. Fucoxanthin-containing supplements are commercially available, and some human studies have investigated their potential effects on body fat and liver function markers [162,163,164,165].

### 5.5. Cardioprotective Effects

Cardiovascular diseases remain one of the leading causes of death worldwide, and according to data from the World Health Organization (WHO), they account for approximately 31% of global deaths each year. In the treatment of cardiovascular diseases, there is a growing focus on diet, with particular emphasis on the natural compounds found in food [123]. Algae, due to their richness in biologically active compounds—including omega-3 fatty acids, astaxanthin, polysaccharides, biologically active peptides, and antioxidants—exhibit various beneficial effects on the cardiovascular system [166]. EPA and DHA fatty acids produced from microalgae exhibit hypolipidemic effects, leading to a reduction in blood triglyceride levels and systolic blood pressure in humans; they may also play a role in the prevention of cardiac arrhythmias [167,168].

The potential impact of algae on cardiovascular disease risk factors has also been evaluated in clinical studies. In a randomized, placebo-controlled trial involving 34 adults who were overweight or obese and had elevated LDL cholesterol levels, 12 weeks of supplementation with an extract from the brown alga *Fucus vesiculosus*—rich in polyphenols—was associated with an approximately 9.5% increase in HDL cholesterol levels compared to the placebo group. However, no significant differences were observed in other markers assessed, including total cholesterol, triglycerides, glucose, or markers of inflammation. These findings suggest that the effect of algal extracts on cardiovascular risk factors may be limited and require confirmation in larger clinical trials [169]. Fucoidans are sulfated polysaccharides found in the cell walls of brown algae [170]. Many authors point out that numerous species of brown algae, including *Padina gymnospora* and *Hormophysa cuneiformis*, are potential sources of natural anticoagulants [170]. Furthermore, polysaccharides derived from the brown alga *Padina tetrastromatica* exhibit anticoagulant activity by inhibiting platelet aggregation in in vitro studies and in vivo studies in animals [171]. In turn, polysaccharides from the alga *Porphyra yezoensis* exhibit hypocholesterolemic effects in animal studies, lowering LDL cholesterol levels and increasing HDL cholesterol levels [172]. Furthermore, sulfated polysaccharides derived from green algae, such as ulvan and sulfated rhamnans, may exert cardioprotective effects through several mechanisms. Ulvan may improve lipid profiles by modulating the expression of lipid metabolism-related receptors, arnesoid X receptor (FXR) and the peroxisome proliferators-activated receptor γ, as demonstrated in animal studies [173]. In contrast, sulfated rhamnans have demonstrated thrombolytic and anticoagulant activities in in vitro studies and in vivo animal models, suggesting their potential role in preventing thrombosis and supporting vascular health [174].

Algae are valued for their high content of various antioxidants, including carotenoids, polyphenols, and vitamin C, which inhibit the development of atherosclerosis and cardiovascular diseases by reducing oxidative stress [166]. Clinical studies indicate that the combination of astaxanthin and DHA may enhance antioxidant protection, reduce lipid peroxidation, and lower MDA levels, as well as having a beneficial effect on arterial elasticity and blood pressure [175]. The antioxidant function of astaxanthin may significantly reduce the risk of myocardial infarction by minimizing damage to heart tissue [176].

### 5.6. Neuroprotective Effects

The protection of nerve cells against damage is an important aspect of the action of bioactive compounds. Importance is attached in this regard to the reduction in oxidative stress, to which the central nervous system is especially susceptible due to its high oxygen demand and high lipid content. Excessive production of ROS can lead to lipid peroxidation, protein and DNA damage, and impaired neuronal function; therefore, the antioxidant properties of algae may constitute one of the mechanisms of neuroprotective action [177].

Algae-derived polysaccharides, pigments, and secondary metabolites have been investigated for their potential neuroprotective effects, particularly in relation to oxidative stress, neuroinflammation, protein homeostasis, and synaptic function [178]. Among polysaccharides, porphyran deserves special attention, as it has demonstrated neuroprotective effects in experimental studies, including by reducing microglial activation and the neuroinflammatory response. Its potential effect on the cholinergic system has also been demonstrated, which may be important for maintaining normal cognitive function [179,180,181].

Pigments found in red algae are believed to play a significant role. Carotenoids, such as lutein and zeaxanthin, found, among other sources, in red algae, may support neuronal protection through their antioxidant and anti-inflammatory effects and by modulating acetylcholinesterase activity [182]. In turn, fucoxanthin, characteristic primarily of brown algae, exhibits strong antioxidant properties, and its metabolites—including fucoxanthinol—may reduce lipid peroxidation and support the activity of antioxidant enzymes. It is reported that carotenoids may currently represent a promising group of compounds in the context of supporting neuronal function and mitigating changes associated with Alzheimer’s disease. Phycobiliproteins, such as phycoerythrobilin and phycocyanin, which are characteristic of red algae, also constitute an important group of pigments. They demonstrate the ability to scavenge free radicals, reduce ROS levels and lipid peroxidation, and protect cells from oxidative damage and apoptosis in in vitro studies [183]. For example, phycoerythrobilin extracted from *Porphyra* sp. also exhibited strong antioxidant activity after in vitro digestion, indicating the significant role of these pigments in the protection of nerve cells [184]. However, evidence from human studies remains insufficient to determine whether algae-derived carotenoids can prevent or slow the progression of Alzheimer’s disease, limiting the extent to which findings from animal models can be extrapolated to humans [185]. Phenolic compounds also represent an important group of neuroprotective agents; their ability to neutralize free radicals may limit oxidative damage to nerve cells. The potential ability of bromophenols isolated from red algae to inhibit acetylcholinesterase activity suggests the possibility of a multifaceted effect on nervous system function.

The diverse biological activities reported for compounds from red algae support further investigation of their potential neuroprotective properties. Nevertheless, their suitability as therapeutic agents for neurodegenerative diseases, including Alzheimer’s disease, remains unconfirmed and requires further preclinical and clinical research [186,187,188,189].

### 5.7. Anticancer Effects

Experimental studies suggest that selected algae-derived compounds may affect cancer cell proliferation, survival, and processes associated with the tumor microenvironment. Moreover, although numerous in vitro and in vivo studies highlight the promising anticancer properties of macroalgae, clinical studies remain limited, with substantial variability in anticancer efficacy and toxicity observed across different species and even among individuals of the same species [190]. In animal studies, chlorophylls a and b derived from green algae may protect DNA against damage and mutations caused by carcinogenic agents [190]. Syphonaxanthin, also present in green algae, exhibits cytotoxicity toward colorectal cancer cells in in vitro studies [191]. Furthermore, in vitro studies indicate that ulvans derived from green algae exhibit antiproliferative activity and induce apoptosis in various cancer cell lines. However, no clinical studies in humans have been conducted to date, highlighting the need for further clinical research to establish the potential anticancer effects and safety of these compounds in humans [192].

In in vitro studies, fucoxanthin and phlorotannins derived from brown algae have demonstrated potential anticancer activity by inducing apoptosis in cancer cells through mechanisms involving caspase activation [193,194]. Fucoxanthin exhibits potential anticancer activity against colorectal, breast, and prostate cancers; however, its limited bioavailability may hinder its effective use in therapy [195,196]. In contrast, phlorofucofuroeckol A may inhibit processes that promote the survival and proliferation of cancer cells in in vitro studies [197], while fucosterol has been reported to inhibit tumor cell proliferation in mouse models [198]. Furthermore, compounds present in algae, such as polyphenols, carotenoids, lipids, and polysaccharides, can inhibit the growth and migration of cancer cells and limit the formation of new blood vessels necessary for tumor growth [190]. Polyphenolic compounds found in the brown algae *Padina australis* and *Stoechospermum marginatum* exhibit strong antioxidant properties; however, their potential significance in cancer prevention and treatment requires further research and standardization of evaluation methods [199].

An overview of the health-promoting properties of algae and algae-derived bioactive compounds, supported by findings from in vitro and in vivo studies, is provided in Table 2.

## 6. Digestibility, Bioaccessibility, and Bioavailability of Algae-Derived Bioactive Compounds

### 6.1. Protein

The protein digestibility of microalgae depends on factors such as the species, the structure and composition of the cell wall, the presence of anti-nutritional compounds, and the methods used to process the biomass [200]. Certain phenolic compounds, including tannins, can bind to proteins or interfere with the activity of proteolytic enzymes, thereby limiting protein breakdown [201]. The cell wall can also serve as a significant barrier; its composition and resistance to digestion vary between species [202]. In the case of *C. vulgaris*, the cell wall structure—characterized by a high cellulose content—can restrict the access of enzymes to intracellular proteins. The literature sources report a protein digestibility of approximately 70–80% for this species; however, this figure should be interpreted with consideration for the analytical method employed and the specific preparation of the biomass [202]. Conversely, *A. platensis* may exhibit relatively high protein digestibility (80–90%), which is partly due to the different structure of its cell envelopes, including a low cellulose content [203]. In a study of nine microalgae species, the highest crude protein digestibility (approx. 80%) was observed for *Nannochloropsis sphaeroides* and *A. platensis*, while the lowest values among those reported (approx. 71%) were found for *P. tricornutum* and *Porphyridium purpureum* [200]. These results indicate interspecies differences in protein digestibility, which are significant when selecting microalgae for nutritional applications [204].

Protein digestibility can also be modified using appropriate pretreatment methods, which disrupt cell structures and facilitate the access of digestive enzymes to intracellular components. Studies have shown that ball milling increased the apparent protein digestibility of *Microchloropsis gaditana* to 62–78% [205], whereas for biomass from *Arthrospira*, *Chlorella*, *Nannochloropsis*, and *Phaeodactylum*, crude protein digestibility reached 78–84% [206]. Following high-pressure homogenization, the apparent protein digestibility of *C. vulgaris* reached 77–87% [207].

The bioaccessibility of microalgal protein depends on the species, as well as the conditions under which the biomass is harvested and processed. In various studies, these values ranged from 60–74% for commercial *Chlorella* and *Auxenochlorella* biomass, while for laboratory-cultivated *C. vulgaris*, the figure was 43%. These differences indicate that the origin and properties of the biomass can influence protein release during digestion, thereby affecting its availability for potential absorption [208,209].

Research on bioaccessibility is complemented by analyses of protein bioavailability conducted using animal models. In mice fed a diet containing 5–25% biomass of *C. vulgaris*, *Nannochloropsis oceanica*, or *P. tricornutum*—subjected to cell disruption via a ball mill—the apparent biological value of the protein was 58–77%, a result comparable to that of the control diet [210].

### 6.2. Lipids

The digestibility of lipids from microalgae depends on the species and the fatty acid profile. In a study on rainbow trout, whose diet included 30% biomass of either *I. galbana* or *Tetraselmis chui*, lipid digestibility ranged from approximately 57% to 77%; the lower values observed for *I. galbana* were attributed to its high content of SFAs. At the same time, PUFAs exhibited the highest digestibility (up to 91.7%), while SFAs showed the lowest (58.9%). Additional mechanical comminution and enzymatic treatment did not significantly improve digestibility compared to freeze-drying alone [211].

Furthermore, in vitro studies indicate limited bioaccessibility of lipids from algal biomass. For *I. galbana*, it was 7–15% [212], while for *C. vulgaris*, in an infant digestion model, it was only 2.5% [213]. In the case of *N. oculata*, the bioaccessibility of fatty acids reached 34% following a change in pH from 7.0 to 10.0 [214]. Meanwhile, in the brown alga *F. spiralis*—which contain 3.5% lipids by dry weight—the bioaccessibility of lipids was approximately 12.1%, and that of fatty acids was 13.0% [15]. The limited release of lipids may stem from their association with cell membranes and the presence of polysaccharide-rich cell walls, which hinder the breakdown of biomass during digestion [215].

The digestibility and bioaccessibility of lipids are also influenced by the pretreatment method employed. High-pressure homogenization of *Nannochloropsis* biomass increased lipid digestibility to 40–62% and the bioaccessibility of EPA to 13–29% [209]. Meanwhile, ball milling of *Microchloropsis gaditana* biomass increased the apparent digestibility of lipids to 50–82% [205].

Furthermore, a randomized clinical trial involving 74 adults demonstrated that the bioavailability of DHA and EPA from microalgal oil supplements—assessed based on their levels in plasma phospholipids after 6 and 14 weeks of supplementation—was non-inferior to that from fish oil. These findings indicate that microalgal oil can serve as a bioavailable source of long-chain omega-3 fatty acids, although it is important to distinguish between studies on the digestibility of whole biomass and those concerning the bioavailability of isolated oil [216].

### 6.3. Polysaccharides

Dietary fiber and polysaccharides constitute a significant portion of algal biomass, typically accounting for 33–62% of the dry weight in macroalgae [215]. Most of these compounds are not broken down by human digestive enzymes, and their properties depend on factors such as chemical composition, solubility, viscosity, and fermentability [215]. In an in vitro study, the total dry weight digestibility was approximately 75% for *Chlorella pyrenoidosa* and 94% for *A. platensis* [217]. In another study, the fiber content in *C. vulgaris* and *A. platensis* was found to be approximately 6% [218].

Furthermore, marine algal polysaccharides exhibit prebiotic potential, as they can be fermented by the gut microbiota. In vitro studies have demonstrated the degradation of *Ascophyllum nodosum* polysaccharides by human fecal microorganisms [219], as well as the gradual depolymerization of polysaccharides from the red macroalga *Neoporphyra haitanensis* during fermentation [220].

Algal polysaccharides are relatively resistant to digestion in the upper gastrointestinal tract, allowing them to reach the large intestine, where they can be fermented by microbiota, thereby exhibiting potential prebiotic effects [221].

### 6.4. Pigments and Polyphenols

#### 6.4.1. Carotenoids

Bioaccessibility of carotenoids refers to the portion of their total content in food that is released from the food matrix during digestion in the upper gastrointestinal tract. Bioavailability, on the other hand, relates to the extent to which carotenoids reach the circulatory system. Both processes depend on the release of carotenoids from the food matrix, their physicochemical properties, and their interactions with other food components [222].

The bioaccessibility of carotenoids varies and is often relatively low [222]. It is assessed using in vitro studies that simulate gastric and intestinal digestion, such as the standardized INFOGEST method [223]. The structure of the food matrix—including the presence of cell walls, which can limit the release of carotenoids—has a significant impact on this process. Mechanical disruption of cells and the addition of lipids can enhance the release and transfer of compounds into the micellar fraction, thereby facilitating their availability for absorption [222,224]. Conversely, dietary fiber and certain mineral ions can limit micelle formation, thereby reducing the bioaccessibility of carotenoids [225,226].

In vitro studies have demonstrated significant differences in the bioaccessibility of individual carotenoids. For fucoxanthin, it was 11.4%, while for fucoxanthinol, it was 21.3%. The use of nanoemulsions increased the bioaccessibility of fucoxanthin to 33.9–46.7% [227,228]. The bioaccessibility of β-carotene, on the other hand, depended on the microalgal species, ranging from 0–2.6% for *Chlorella* sp. to 10% for *Chlamydomonas reinhardtii* and 29% for *P. tricornutum*. These differences have been attributed, among other factors, to the structure of the cell walls, with mechanical cell disruption facilitating the release of carotenoids [229,230,231]. 

#### 6.4.2. Chlorophylls

The bioaccessibility of chlorophyll pigments also depends on the algal species, the method of biomass preparation, and the form of the product. A study involving *Tetradesmus obliquus* evaluated an isolated chlorophyll extract, wet biomass subjected to ultrasound treatment, and whole dried biomass, using in vitro digestion according to the INFOGEST protocol and a Caco-2 intestinal cell model. For the isolated chlorophyll extract, a value of 33.5% was recorded, highlighting the importance of the form in which the pigment is delivered [232]. In studies on *Chaetoceros calcitrans*, the relative bioaccessibility of the total chlorophyll fraction was 12.22%, with pheophytin a being the only detectable bioavailable chlorophyll, reaching a bioavailability of 15.01%. This value was higher than that observed for *Scenedesmus obliquus* biomass subjected to ultrasound treatment (approximately 10%). The total bioavailability of chlorophylls from *C. calcitrans* was also higher than that from sonicated *S. obliquus* biomass (3%), but lower than that from isolated microalgal extracts (approximately 33%) [233].

#### 6.4.3. Phenolic Compounds

Research on the bioaccessibility of phenolic compounds derived from algae is limited, and available findings indicate variations depending on the algal species and the type of matrix. In the case of the macroalga *Sargassum wightii*, values of 39.91% for total polyphenol content (TPC) and 25.64% for antioxidant activity (FRAP) were recorded following simulated in vitro digestion [234]. For freeze-dried *F. spiralis*, these values were 22.4% and 59.5%, respectively [235]. It should be noted, however, that TPC and FRAP values reflect total polyphenol content and antioxidant activity, rather than direct absorption by the body. An increase in these values following digestion may be attributed to the release of phenolic compounds from the algal matrix, as well as changes occurring under the conditions of simulated digestion [234]. In studies of commercial *C. vulgaris* microalgae preparations—available as powders and tablets—the bioaccessibility of phenolic compounds (including *p*-coumaric acid, cinnamic acid, and kaempferol 7-rhamnoside) was found to be 1–2% following incubation in simulated digestive juices [236].

Given the limited number of available studies, it is difficult to definitively determine the bioaccessibility of polyphenols derived from algae. Further research is required to better characterize the release of these compounds during digestion, as well as their actual absorption within the body [234].

## 7. The Application of Algae in Food Technology

In recent years, there has been growing interest in the use of algae in food technology, primarily due to their rich chemical composition and the presence of numerous compounds with potential health-promoting properties. The use of algae in food can help meet the growing demand for protein. Furthermore, these organisms provide many valuable bioactive substances, such as amino acids, PUFA, vitamins, and minerals, making them a valuable addition to conventional food products [237]. Algae and their processing byproducts can be used to develop new food products [238]. Furthermore, algae can be used as natural food additives, including as thickeners and gelling agents, to achieve the desired functional properties of products [239].

### 7.1. Functional Foods

The growing interest in healthy and plant-based foods is contributing to the increasingly widespread use of algae as a functional ingredient. Microalgae due to their high protein content, can be a valuable addition to many food products [240]. Algae can serve as a valuable ingredient in functional foods, thanks in part to their content of omega-3 fatty acids, including DHA and EPA [218], natural pigments—carotenoids and xanthophylls [241], vitamin B12 [242], and minerals—iron, zinc, selenium, calcium, and potassium [215]. Furthermore, their distinctive flavor makes them suitable for use in products that serve as alternatives to fish and seafood. The bioactive compounds found in algae can positively influence the nutritional value and health-promoting properties of food products [243]. Algae, as a food additive, are valued among vegans because they enable the production of foods with desirable functional and organoleptic properties. Due to their high protein content (up to 70%) and high protein digestibility (up to 95%), especially *Spirulina* and *Chlorella*, they are used in the production of meat substitutes [244]. In turn, algae-based flours and lipid powders are used in vegan cooking as egg substitutes [245].

#### 7.1.1. Grain Products

Grain products are also of interest when it comes to enriching food products with algae. These products are compatible with many bioactive compounds and serve as an effective carrier for health-promoting ingredients [246]. Algae of the species *Ulva lactuca* and *Gelidium corneum* significantly increased the antioxidant activity (by increasing the content of polyphenols and flavonoids) as well as the protein content of sourdough bread [247]. In turn, Tertychnaya et al. [248] enriched bread with 5–10% *Dunaliella salina* biomass, achieving an increase in protein content from 7.1% in the control sample to 8.4–9.3% in the enriched products, without compromising their basic physicochemical and sensory properties. Algal biomass is used in many baked goods, including bread, cookies, breadsticks, and crackers, primarily to increase their nutritional value [249]. The addition of various microalgal species, such as *Arthrospira*, *C. vulgaris*, *Nannochloropsis*, or *Tetraselmis*, can increase protein and mineral content, as well as affect the physicochemical and sensory properties of the products [250,251].

Microalgae affect the rheological properties of bread dough by increasing water absorption, limiting gluten network development, and reducing stability during mixing, with the effect depending on the species and level of addition [252]. *C. vulgaris* exhibits a stronger effect; an addition of up to 3% can improve the viscoelastic properties of the dough, whereas higher concentrations have an adverse effect on its rheology [253,254]. *A. platensis* has a milder effect, increasing the dough’s elasticity while simultaneously reducing its extensibility [6,255]. In turn, cookies may serve as a more suitable carrier for functional ingredients derived from microalgae than high-gluten baked goods. Their specific processing characteristics and higher fat and sugar content limit the impact of algal additions on the product’s structure and can mask undesirable off-flavors [256,257]. As a result, cookies enriched with microalgae often exhibit better sensory acceptance than baked goods such as bread [252].

#### 7.1.2. Dairy Products

Globally, there is a downward trend in milk consumption, while fermented dairy products—particularly cheese and yogurt—are growing in popularity among consumers [258]. Today, dairy products serve as a vehicle for nutritional and functional innovations, and in this context, the incorporation of algae into dairy matrices can positively impact the nutritional value and functional quality of the product (Table 3) [259,260].

The dairy products most enriched with algae are yogurts, cheeses, and ice cream, and the main goal of fortification is to increase protein content and provide biologically active components [261]. Fermented dairy products are most often enriched with algae prior to the fermentation process, as their contact with lactic acid bacteria is critical to product quality [262]. Yogurt is a dairy product that has been particularly extensively studied in terms of algae application. The inclusion of algae in yogurt improves not only its nutritional profile but also its functional properties—the ability to form gels, emulsify, and stabilize yogurt ingredients promotes the production of products with a smoother, creamier texture and greater stability [263,264]. Pleasant sensory experiences correlate with technological benefits in yogurt production and processing [265]. It is also becoming increasingly common to fortify cheese, a practice that has been shown to modify both its nutritional value and certain physicochemical properties. Faustino et al. [266] were among the first to study the use of the red alga *Osmundea pinnatifida* in cheese production. The introduction of algae into the cheese matrix led to an increase in protein and carbohydrate content, while the pH value decreased slightly—from 5.95 in the control sample to 5.61 in the enriched product. A similar trend toward increased protein content was observed in cheeses enriched with the microalga *C. vulgaris*. Yilmaz-Ersan and Suna [267] demonstrated a significant increase in protein content even with a 1% addition of *C. vulgaris*, accompanied by a reduction in lipid content. In turn, Mohamed et al. [268], using 1%, 2%, and 3% *C. vulgaris*, also noted an increase in protein and carbohydrate content, along with a reduction in lipid and ash content and a decrease in pH.

**Table 3:** The use of algae in dairy products and their impact on technological and nutritional properties.

Product	Algal Species	Addition Level	Technological/Nutritional Function	Reference
Ayran	*Chlorella vulgaris*	0.25%	30-min reduction in fermentation time; 43% and 34% increase in fat and protein content; increased product viscosity	[269]
Dairy curd	*Nannochloropsis salina*	Whole algae—14.2%; ruptured algae—7.5% dry matter	slowing of gelation and modification of curd microstructure; increase in EPA content	[270]
Labneh	*Athrospira plantesis*	0.2, 0.4, 0.7 i 1% *w*/*v*	increased probiotic viability compared to the control sample; increased fiber content (1.41–2.57%); increased content of protein, zinc, iron, potassium, and magnesium (*p* < 0.05); enhanced bioactive properties (increased levels of polyphenols and vitamins)	[271]
Yogurt spread	*Athrospira plantesis*	1.5–3.5%	increased content of protein, total phenolics, and carotenoids	[263]
Yogurt	*Isochrysis galbana*	2% *w*/*w*	increased protein content; increased fracture, hardness, cohesiveness, springiness, gumminess, and chewiness in fresh and rehydrated yogurts	[237]
Freeze-dried yogurt	*Athrospira plantesis*	0.5%	increase in dry matter content and reduction in syneresis; protection of lactic acid bacteria against the effects of freeze-drying; beneficial effect on the survival of lactic acid bacteria	[272]
Ice cream	*Athrospira plantesis*	0.6% i 1%	lowering the melting point of ice cream; increase in protein content,	[273]

#### 7.1.3. Snacks

Microalgae can serve as an interesting ingredient to enrich energy bars, enhancing their nutritional value and functional potential. *A. platensis* and *C. vulgaris* are distinguished by their high content of complete protein—which includes all essential amino acids—as well as the presence of vitamins, minerals, phenolic compounds, pigments, sterols, and PUFAs. Their inclusion in bar formulations can therefore simultaneously increase the protein content and levels of selected micronutrients while enriching the product with compounds that have antioxidant potential. Of particular interest is the presence of vitamin B12, which further highlights the potential for using microalgae in the development of bars with enhanced nutritional value [274].

Furthermore, microalgae can serve as an interesting alternative to synthetic dyes used in the food industry. Studies have demonstrated the potential to use *C. vulgaris* biomass to color baked goods, such as brioche, as well as extracts from *Tetraselmis chuii* to color fondant. The addition of 1% *C. vulgaris* biomass produced a color like that of an egg-containing product, while a hydroethanolic extract from *T. chuii* imparted an intense green color to the fondant at a concentration as low as 0.05%. The use of extracts also helps to reduce the characteristic taste of microalgae. The results indicate that microalgae, due to the presence of natural pigments such as chlorophylls and carotenoids, can be used as natural colorants in various food products, offering a potential alternative to synthetic colorants [275].

Algae can be used as an ingredient in various types of snacks, such as crackers and extruded products [106]. The addition of 6% *A. platensis* and *C. vulgaris* increased the protein content to 13.2–13.5%, while crackers containing *A. platensis* exhibited the highest antioxidant activity and the best sensory acceptance [106].

### 7.2. Algae as Natural Food Additives

In addition to enriching foods with nutrients, there is growing interest in the use of algae as ingredients that serve technological functions in food production. Phycocyanin, a natural protein derived from algae—primarily cyanobacteria—that exhibits antioxidant and anti-inflammatory properties, can be used not only as a bioactive substance but also as an ingredient in food preparations. Another example of a compound derived from algae is κ-carrageenan, a saccharide found in red algae, used as a biopolymer with thickening and gelling properties. The combination of phycocyanin with κ-carrageenan enables the production of stable dispersions, such as nanoemulsions. The use of these natural ingredients improves the physical stability of products while providing an alternative to some synthetic stabilizers [276]. Other studies have shown that proteins extracted from the microalga *Nannochloropsis gaditana* can act as natural bioemulsifiers, stabilizing emulsions used in the food industry. Their properties depend, among other factors, on protein concentration and environmental conditions such as pH, temperature, and salt concentration [277]. Pehlivanow et al. [278] point out that the green alga *A. plantesis* can be used in the food industry as a natural stabilizer and emulsifier, serving as an alternative to synthetic substances. Its use also makes it possible to enrich food products with nutrients.

Algae can be used as natural ingredients that shape the aroma and flavor of food products. The volatile compounds they contain are responsible for characteristic aroma notes, including marine, plant-based, fresh, grassy, and umami notes [279]. However, the aromatic profile of algae can vary depending on the species, storage conditions, and the processing methods used. The use of algae as flavoring ingredients is possible in many food groups, including pasta, bread, dairy products, snacks, and meat and fish products [279]. For example, adding *U. pinnatifida* (wakame) to pasta can impart a mild marine aroma [280], while the addition of *A. platensis* affects the aroma profile of bread and may result in the emergence of characteristic algal notes [250]. In meat and fish products, algae can enhance the flavor and introduce marine and spicy notes. Algal concentration is a key factor. Small amounts can enhance a product’s sensory profile, whereas too high a concentration can lead to the development of an intense, fishy, marine, or bitter aroma, reducing consumer acceptance. Therefore, when developing products containing algae, it is essential to select the appropriate species, form of the raw material, and its quantity, as well as to control the manufacturing process [279].

Due to their strong antioxidant and antimicrobial properties, algae are used in food as natural preservatives. Carotenoids such as β-carotene, astaxanthin, lutein, and fucoxanthin are of particular importance, as they can limit oxidative processes occurring in food. β-carotene, obtained from sources such as *D. salina* and *Tetradesmus almeriensis*, is used as a natural antioxidant and food coloring [281,282,283]. A particularly interesting compound is astaxanthin, which is sourced from, among others, *Haematococcus pluvialis* and *Chlorella zofingiensis*. It is characterized by very strong antioxidant activity, which, according to the cited studies, may be up to ten times greater than that of other carotenoids [284]. Lutein, obtained from sources such as *A. platensis* and *Chlorella* species, also exhibits preservative potential. Its use can increase the levels of antioxidants in food products, thereby supporting their oxidative stability. Fish products are a good example; in these, the addition of lutein can help extend shelf life and increase the product’s nutritional value [285]. The antioxidant properties of microalgae also stem from the presence of vitamins C and E, as well as glutathione. Vitamin C participates in the neutralization of ROS and the regeneration of other antioxidants, while vitamin E limits lipid peroxidation by interrupting oxidative chain reactions [286,287]. Glutathione, in turn, aids in the removal of ROS and participates in the regeneration of vitamins C and E. Thanks to the synergistic action of these compounds, the antioxidant system of microalgae can effectively limit processes leading to food quality deterioration [288,289]. Another group of compounds with potential preservative applications consists of phenolic compounds. They can inhibit the oxidation of lipids and proteins, thereby limiting the degradation of food components. Their content depends on the microalgal species and cultivation conditions. Studies have shown, among other things, high levels of phenolic compounds in *Neochloris oleoabundans* and *Wilmottia murrayi*, exceeding 20 mg of gallic acid equivalent per gram of biomass [290].

### 7.3. Technological and Sensory Properties of Microalgae-Enriched Foods

In addition to their nutritional value and potential health-promoting properties, algae can influence the technological and sensory characteristics of food products. This effect depends on the microalgae species, the form in which they are used (e.g., biomass, powder, or extract), the concentration of the additive, and the type of food matrix [252]. Consequently, while enriching food with microalgae can improve certain product attributes, it may also present challenges regarding consumer acceptance [291].

Texture and rheological properties are key parameters to consider when developing algae-enriched products. Algal polysaccharides can modify the consistency and stability of dairy products, while natural pigments can alter their color and appearance [292]. One study demonstrated that the type of flour and the addition of microalgae affected the rheological properties of the batter—including the consistency coefficient (K)—as well as the porosity and hardness of muffins. Incorporating 1.5% microalgae into a blend of spelt and wheat flour increased the consistency coefficient of the batter and the porosity of the muffins, while simultaneously reducing their hardness compared to products made solely from wheat flour. Despite these differences, all the products tested were accepted by consumers in terms of appearance, taste, and overall quality [293]. In a study by Masino et al. [294] it was shown that the addition of Spirulina at a level of 1–2% influenced the color, volatile compound profile, and sensory properties of bread. Darker crust and crumb were observed, and at a 1% inclusion level, a significant reduction in hardness was also noted compared to the control sample. Sensory analysis revealed an intensification of bitter and marine notes, while other measured texture parameters did not undergo significant changes.

Due to their sodium content, algae have a salty taste, which allows them to be used as a substitute for table salt. It is estimated that 1 g of table salt is equivalent to 9–18 g of dried algae. Amino acids present in algae, such as glutamate and aspartate, are also responsible for the characteristic umami taste and enhance the flavor profile of food products to which they are added [295]. Furthermore, lipid oxidation products, such as aldehydes, contribute to the distinctive aroma of algae, which includes grassy, fatty, and fishy notes [296].

Because of their characteristic marine taste and aroma, algae can be used in the production of fish and shellfish analogues [243]. For instance, they have been used to develop plant-based fish cakes inspired by a traditional Portuguese cod dish. These cakes received high sensory acceptance, thanks to their pleasant flavor and a marine aftertaste reminiscent of fish-based products [297]. In other products, such as pasta, their inclusion has had a negative impact on sensory acceptability, diminishing the product’s aroma, taste, and overall acceptance [298].

Changes in specific characteristics of food products—such as texture, rheology, color, taste, and aroma—depend on the species of algae used, their concentration, and the form in which they are added (e.g., as biomass, powder, or extract). The type of food matrix is also significant, as individual ingredients within a product may interact with algal components in different ways. These factors influence both the technological properties and sensory quality of the final product, as well as its overall consumer acceptance [243,299].

## 8. Safety and Toxicological Aspects of Algae

### 8.1. Heavy Metals

One of the limitations of using seaweed in food is its potential to accumulate heavy metals, including arsenic, cadmium, lead, and mercury. Their content depends primarily on the species and environmental conditions; therefore, it is essential to monitor contamination levels in algae-based products [300]. When incorporating algae into food products, it is necessary to quantitatively monitor arsenic levels in the fortified products. Inorganic forms of arsenic can damage DNA, including increasing the incidence of lung, bladder, and kidney cancer [301,302]. The arsenic content in seaweed varies by species, with most occurring in the form of arsenosaccharides, and the content of toxic inorganic arsenic is typically low [300]. An exception is *L. digitata*, which is characterized by elevated levels of inorganic arsenic [303]. Among seaweed-based products, hijiki contains the highest levels of inorganic arsenic [304]. The remaining products—arame, wakame, kombu, and nori—are considered safe for consumption and contain inorganic arsenic concentrations below 0.3 µg/g [300]. However, it is estimated that the bioavailability of inorganic arsenic in heat-treated seaweed products is up to 15% [8].

A potential risk associated with consuming algae may be excessive intake of minerals found in sea salt, such as bromine. It has been suggested that bromine intake may cause damage to the nervous system, DNA, and organs in mammals. It is reported that bromine concentrations in the urine of women living in Korea, China, and Japan correlate with their consumption of seafood, the primary source of microalgae. It should be noted, however, that the observed correlations may also have been influenced by the consumption of plant products grown in areas where methyl bromide was used. This is because its use can lead to a significant increase in the body’s total exposure to bromine. This underscores the importance of considering all potential sources of exposure to this element when assessing the effects of algae consumption on the body [8].

### 8.2. Allergenicity

To date, few studies have been published on the allergenic properties of algae. Potential allergens in algae include, among others, phlorotannins, polysaccharides, and proteins [305]. The most reported allergic reactions to algae involve *Spirulina* and *Chlorella*, which are widely used in dietary supplements. Studies have shown that both species contain proteins capable of triggering allergic reactions. Several potential allergens have been identified in *Chlorella*, while in *Spirulina*, the *C*-phycocyanin protein, among others, has been detected, which may have allergenic properties [305]. Studies evaluating the allergenic potential of carrageenan derived from algae have shown that 20% of study participants tested positive for immunoglobulin E reactivity to this ingredient [306]. Characteristic symptoms of an allergy to carrageenan derived from the algae *Porphyra*, *C. crispus*, and *P. palmata* include hives, angioedema of the face, tingling of the lips and tongue, a tight sensation in the throat, and a stuffy nose [307].

Some proteins found in seaweeds, such as *Ulva*, *Porphyra* (nori), and *Saccharina*, are similar to known allergens found in fish, crustaceans, and mollusks. This means that seaweed may trigger allergic reactions in people who are allergic to these products. It is also possible that the allergens in seaweed originate from contamination by marine organisms. However, further research is needed to accurately assess this risk [308,309].

However, there have been cases of poisoning following the consumption of products containing algae. A case of anaphylactic shock was reported in a young man after he ate a spirulina tablet; however, the purity of the spirulina consumed and its source are unknown [310].

### 8.3. Toxins

Some red algae may contain compounds with potentially toxic effects. One example is kaempferic acid, found, among others, in dulse (*P. palmata*) and *Digenia simplex*. Due to its structural similarity to glutamate, it has neurotoxic effects at high doses. However, concentrations capable of damaging neurons are significantly higher than those typically found in dulse, although clear safety standards for humans have not yet been established. The content of kaempferic acid can also vary significantly among different algae species [8].

Other compounds present in red algae also pose a potential risk [311]. Certain species of *Gracilaria* and *Caulerpa*, consumed as sea vegetables, have caused cases of poisoning and, in isolated instances, even deaths, when toxic species were ingested or mistakes occurred during harvesting. One example is *Gracilaria edulis*, whose consumption has been linked to the presence of toxic polycavernosides [8].

Some microalgal species can produce toxins. Many environmental factors, including temperature, light intensity, and nutrient availability, can contribute to the formation of harmful algal blooms [312]. The increase in the number of toxic microalgae during blooms leads to the release of harmful substances, such as saxitoxin and nodularin, into the water. These toxins can accumulate in the food chain and pose a threat to human health, causing, among other things, neurological, gastrointestinal, cardiovascular, and respiratory problems [313,314]. Toxins can enter the human body in three ways: through the consumption of contaminated food, inhalation of toxins in aerosol form, and through skin contact with contaminated fluids [315].

## 9. Future Perspectives for Alga; Production and Utilization

Despite the great potential of microalgae in sustainable food production, their wider use requires further research and technological development. Future areas of development include, among others, increasing production efficiency, reducing costs, utilizing new sources of nutrients, and developing applications for microalgal biomass in various industries [316].

### 9.1. Optimization of Algal Cultivation and Production

One of the key areas of development remains the optimization of algal cultivation conditions. It is essential to better understand the impact of light intensity and quality, temperature, nutrient availability, pH, salinity, and carbon dioxide concentration on the growth of individual species and the accumulation of desired metabolites [317]. Research into the use of automated monitoring and control systems, which allow for the dynamic adjustment of culture parameters to the current state of the biomass, is particularly promising. The development of such solutions may contribute to increased production stability and reduced raw material losses [318,319]. Artificial intelligence and machine learning could significantly improve microalgae cultivation and the production of valuable bioactive compounds in the future. These technologies enable real-time monitoring of cultivation conditions, prediction of changes in the process, and rapid response to adverse deviations. At the same time, analysis of data on metabolic pathways and gene regulation may allow for better optimization of microalgal growth conditions, increasing the yield of biomass and desired metabolites. The development of these technologies may therefore facilitate the automation and scaling of microalgae production at the industrial level [320].

### 9.2. Algae in the Circular Economy

Another important area of research is the use of molecular biology methods, metabolic engineering, and omics tools. Analysis of genomes, transcriptomes, proteomes, and metabolomes may enable a more precise determination of the mechanisms responsible for biomass growth and the synthesis of specific metabolites [321]. For example, genomic data resources have been developed for microalgae of the *Nannochloropsis genus*. Their availability has enabled a better understanding of the genome organization, genetic composition, and molecular mechanisms characteristic of these microalgal groups [322]. In the long term, this may lead to the development of strains with increased productivity or the targeted production of selected compounds, such as lipids, proteins, pigments, polysaccharides, or substances with biological properties [321,323].

### 9.3. The Use of Algae for Wastewater Treatment

Research on the use of algae in a circular economy will also be of great importance, as it can contribute to better resource utilization and a reduction in greenhouse gas emissions. Of particular importance is the use of microalgae for CO_2_ capture and the recovery of nutrients from waste [324]. An interesting approach is combining biomass production with wastewater treatment and the recovery of biogenic components. Algae can utilize nitrogen and phosphorus compounds present in wastewater streams, converting them into biomass that can then be subjected to further processing [325]. This helps reduce the consumption of freshwater and synthetic nutrients, thereby lowering cultivation costs and increasing the profitability of further biomass utilization [326].

### 9.4. Future Potential of Algal Biomass

Future prospects for algae utilization are closely linked to the development of sustainable and economically viable biorefinery concepts. The comprehensive use of algal biomass—encompassing the simultaneous production of biofuels and the recovery of high-value products such as proteins, pigments, lipids, carbohydrates, vitamins, antioxidants, and bioactive peptides—can enhance the economic viability of algae-based technologies [327]. In the future, the development of efficient biomass extraction and processing methods, the integration of multiple processes within a single biorefinery, and the optimization of energy and resource consumption will be of particular importance. Such an approach could increase the value of algal biomass while fostering the development of sustainable algal applications across the food, pharmaceutical, cosmetic, and energy sectors [328].

## 10. Conclusions

Algae represent a promising source of biologically active compounds with significant potential in human nutrition and food technology. Both microalgae and macroalgae contain a wide array of valuable constituents, including proteins, bioactive peptides, polyunsaturated fatty acids, pigments, polysaccharides, vitamins, minerals, and compounds with antioxidant properties—such as polyphenols and carotenoids. The diversity of these components accounts for a broad spectrum of observed biological activities, including antioxidant, antimicrobial, anti-inflammatory, antihypertensive, and antidiabetic effects, as well as an influence on lipid metabolism.

The use of algal biomass and the bioactive compounds derived from it in food products offers the opportunity to enhance their nutritional value and impart additional functional properties. Particular attention is focused on algae as a source of proteins and bioactive peptides, which can be obtained—for instance, through enzymatic hydrolysis—and subsequently used as ingredients in functional foods. Algal components can also influence key technological characteristics of food products, such as texture, color, stability, and sensory properties. However, this effect depends on the algae species, biomass composition, processing conditions, and the concentration used.

Despite their significant potential, the wider use of algae is associated with a number of limitations. These include, among others, the variability of biomass chemical composition, the potential presence of undesirable compounds or contaminants, sensory limitations, processing costs, and the need to optimize extraction and fractionation methods. Furthermore, although numerous in vitro and experimental studies indicate the beneficial biological properties of algae-derived compounds, there is still a lack of sufficient evidence from well-designed human clinical trials for many of them. Consequently, claimed health-promoting properties should be evaluated by considering factors such as bioavailability, dosage, compound stability, and interactions with the food matrix.

Future research should focus on developing sustainable and economically viable algae biorefinery technologies that enable the simultaneous recovery of multiple high-value products. It will also be crucial to further understand the properties of bioactive peptides and other algae-derived compounds, as well as to standardize methods for their extraction, identification, and biological activity assessment. Such efforts could facilitate the transition from laboratory research to practical, commercial applications within the food industry. In summary, available data indicate that algae constitute a valuable and versatile raw material for the development of functional foods and innovative food ingredients; however, fully realizing their potential requires further technological, toxicological, and clinical research.

## Figures and Tables

**Table 1:** Chemical composition of selected species of micro- and macroalgae (% of dry weight).

Algae Species	Protein (%)	Total Lipids (%)	Carbohydrates (%)	Ash (%)	Reference
Microalgae					
*Dunaliella* sp.	34.17	14.36	14.57	19.29	[60]
*Nannochloropsis* sp.	30.29	21.78	9.62	11.32	[60]
*Scenedesmus* sp.	30.99	15.07	27.66	15.72	[60]
*Spirulina* sp.	55.8	14.2	22.2	7.8	[61]
*Athrospira plantesis*	56.79	8.33	13.60	10.05	[62]
*Chlorella* sp.	55.3	15.7	25.2	5.8	[61]
*Chlorella protothecoides*	48.2	6.9	31.6	N/A	[63]
*Botryococcus braunii*	39.1	24.9	30.6	5.4	[61]
*Porphyridium aeruginium*	31.6	13.7	45.8	8.9	[61]
*Nannochloropsis granulata*	33.5	23.6	36.2	6.7	[61]
*Chaetoceros calcitrans*	40.3	23	37	N/A	[64]
*Isochrysis galbana*	27.10	10.54	34.32	N/A	[65]
*Tetraselmis gracilis*	33.02	7.95	29.96	N/A	[65]
Macroalgae					
*Gracilaria* sp.	16	1.2	76.6	6.1	[66]
*Gracilaria birdiae*	8.03	0.46	73.01	6.05	[67]
*Durvillaea antarctica*	10.79	0.43	54.57	26.06	[68]
*Macrocystis pyrifera*	9.81	0.21	52.71	30.47	[68]
*Lessonia nigrescens*	9.88	0.23	48.36	33.03	[68]
*Ascophyllum nodosun*	8.70	3.62	N/A	30.890	[69]
*Ulva* sp.	9.24	0.38	49.09	31.40	[70]

Abbreviations: N/A—not available.

**Table 2:** Health-Promoting Effects of Algae and Algae-Derived Bioactive Compounds: Evidence from In Vitro and In Vivo Studies.

Health Effect	Algae Species/Compound or Extract Tested	Research Model	Dose/Concentration	Main Findings	Reference
Antioxidant	Algal source not specified/Fucoxanthin	In vivo (Isoprenaline-induced myocardial infarction in Wistar rats)	Fucoxanthin: 50 mg/kg body weight orally for 30 days; isoprenaline hydrochloride: 85 mg/kg body weight, administered subcutaneously on days 29 and 30	Increased antioxidant enzyme levels (SOD, CAT, GPx) and reduced oxidative stress (TBARS) and cardiac injury markers.	[126]
Antioxidant	Algal source not specified/Fucoxanthin	In vivo (ovalbumin-induced asthma in mice)	50 mg/kg	Increased antioxidant enzyme activity, reduced intracellular ROS production and inflammatory cytokine levels in bronchoalveolar lavage fluid.	[127]
Antioxidant	Brown algae/Fucoxanthin	In vivo (LPS-induced ocular inflammation in rats)	Fucoxanthin: 10 mg/kg body weight	Increased SOD activity and reduced MDA levels, inflammatory cell infiltration, and TNF-α levels.	[128]
Anti-inflammatory	*Chlorella vulgaris* (hot-water extracts)	In vivo (mouse model of colitis)	2 g/kg body weight, orally, for 3 weeks	Alleviated colitis symptoms, increased regulatory T cells (Tregs), and modulated gut microbiota and short-chain fatty acid (SCFA) production.	[138]
Anti-inflammatory	*Caulerpa* spp.; caulerpin	In vivo (peritonitis and DSS-induced colitis in mice)	4 and 40 mg/kg; 4 mg/kg was the most effective dose in colitis	Reduced inflammatory infiltration, disease activity, colon shortening, and tissue damage. Decreased TNF-α, IFN-γ, IL-6, IL-17, and NF-κB p65 levels, while increasing IL-10.	[139]
Anti-inflammatory	*Porphyra dioica*, *Palmaria palmata*, *Chondrus crispus*, *Pavlova lutheri*	In vitro (LPS-stimulated human THP-1 macrophages)	3 µg/mL of algal lipid extracts	Reduced pro-inflammatory cytokines IL-6 and IL-8 and downregulated 14 pro-inflammatory genes. Reduced inflammatory signaling and the expression of pro-inflammatory genes.	[141]
Antidiabetic	*Laminaria digitata*, *Undaria pinnatifida*	In vivo (human study)	5 g of algae with 30 g of starch	Reduced postprandial glucose, insulin, and *C*-peptide responses; increased GLP-1 response after *L. digitata* intake and decreased subjective appetite.	[151]
Antidiabetic	*Athrospira plantesis*	In vivo (human clinical trial; 60 patients with type 2 diabetes)	2 g/day for 3 months	Reduced HbA1c, fasting blood glucose, triglycerides, total cholesterol, and LDL-C; slightly increased HDL-C. No adverse events reported.	[152]
Anti-obesity	Fucoxanthin combined with conjugated linoleic acid (CLA), in complex liposomes	In vivo (mice fed a high-fat diet)	Fucoxanthin (FX) at 0.083 mg/kg body weight/day in FX–CLA complex liposomes, administered orally for 6 weeks.	Investigated effects on obesity and AMPK signaling, a pathway involved in energy metabolism.	[160]
Anti-obesity	*Phaeodactylum tricornutum* extract containing fucoxanthin	In vitro (3T3-L1 mouse fat cells) and in vivo mice fed a high-fat diet).	In vivo: 0.1 mg/kg/day fucoxanthin; 0.81, 1.62, or 3.25 mg/kg/day algal extract, administered orally for 6 weeks.	Reduced lipid accumulation in adipocytes, body weight, fat mass and adipocyte size; improved lipid metabolism and markers of liver damage.	[161]
Cardioprotective	*Fucus vesiculosus* polyphenol-rich extract	In vivo (human clinical trial; 34 overweight or obese adults).	2000 mg/day orally for 12 weeks	Increased HDL cholesterol by 9.5%, but no significant changes in LDL cholesterol, total cholesterol, triglycerides, glucose, insulin, or inflammatory markers compared with placebo.	[169]
Cardioprotective	Sulfated polysaccharide isolated from the green algae *Monostroma nitidum*.	In vitro and in vivo (coagulation assays, rat plasma, and mouse and rat thrombosis models).	2.5, 5, or 10 mg/kg body weight, administered by injection.	Prolonged blood clotting time, reduced platelet aggregation and thrombus formation, and enhanced thrombus breakdown. The anticoagulant effect was mainly associated with increased activity of heparin cofactor II and antithrombin III.	[174]
Neuroprotective	*Pyropia yezoensis*—antioxidant peptides derived from phycoerythrin.	In vitro (human liver cells)	1 µg/mL of synthetic peptides	Peptides reduced oxidative stress caused by hydrogen peroxide and increased SOD expression. PBP2 increased Nrf2 activity and SOD levels, helping protect cells against oxidative stress.	[183]
Anticancer	Green algae/Siphonaxanthin	In vitro (human leukemia cells, human umbilical vein endothelial cells and rat aortic tissue).		Siphonaxanthin reduced leukemia cell viability by inducing cell death, inhibited blood vessel formation and reduced mast cell activation.	[190]

Abbreviations: SOD—Superoxide dismutase; CAT—Catalase; GPx—Glutathione peroxidase; TBARS—Thiobarbituric acid reactive substances; ROS—Reactive oxygen species; LPS—Lipopolysaccharide; DSS—Dextran sulfate sodium; Tregs—Regulatory T cells; TNF-α—Tumor necrosis factor alpha; IFN-γ—Interferon gamma; IL-6—Interleukin 6; IL-8—Interleukin 8; IL-10—Interleukin 10; IL-17—Interleukin 17; NF-κB—Nuclear factor kappa B; GLP-1—Glucagon-like peptide-1; HbA1c—Glycated hemoglobin; LDL-C—Low-density lipoprotein cholesterol; HDL-C—High-density lipoprotein cholesterol; CLA—Conjugated linoleic acid; AMPK—AMP-activated protein kinase; FX—Fucoxanthin; PBP2—Phycoerythrin-derived peptide PBP2; Nrf2—Nuclear factor erythroid 2-related factor 2.

## Data Availability

The data presented in this study are available in the article.
